# Peripheral arterial blood pressure monitoring adequately tracks central arterial blood pressure in critically ill patients: an observational study

**DOI:** 10.1186/cc4852

**Published:** 2006-03-08

**Authors:** Mariano Alejandro Mignini, Enrique Piacentini, Arnaldo Dubin

**Affiliations:** 1 Critical Care Unit, Clínica Bazterrica, Buenos Aires, Argentina; 2 Critical Care Unit, Hospital Mutua Terrassa, Terrassa, Spain; 3 Critical Care Unit, Sanatorio Otamendi y Miroli, Buenos Aires, Argentina

## Abstract

**Introduction:**

Invasive arterial blood pressure monitoring is a common practice in intensive care units (ICUs). Accuracy of invasive blood pressure monitoring is crucial in evaluating the cardiocirculatory system and adjusting drug therapy for hemodynamic support. However, the best site for catheter insertion is controversial. Lack of definitive information in critically ill patients makes it difficult to establish guidelines for daily practice in intensive care. We hypothesize that peripheral and central mean arterial blood pressures are interchangeable in critically ill patients.

**Methods:**

This is a prospective, observational study carried out in a surgical-medical ICU in a teaching hospital. Fifty-five critically ill patients with clinical indication of invasive arterial pressure monitoring were included in the study. No interventions were made. Simultaneous measurements were registered in central (femoral) and peripheral (radial) arteries. Bias and precision between both measurements were calculated with Bland-Altman analysis for the whole group. Bias and precision were compared between patients receiving high doses of vasoactive drugs (norepinephrine or epinephrine >0.1 μg/kg/minute or dopamine >10 μg/kg/minute) and those receiving low doses (norepinephrine or epinephrine <0.1 μg/kg/minute or dopamine <10 μg/kg/minute).

**Results:**

Central mean arterial pressure was 3 ± 4 mmHg higher than peripheral mean arterial pressure for the whole population and there were no differences between groups (3 ± 4 mmHg for both groups).

**Conclusion:**

Measurement of mean arterial blood pressure in radial or femoral arteries is clinically interchangeable. It is not mandatory to cannulate the femoral artery, even in critically ill patients receiving high doses of vasoactive drugs.

## Introduction

Invasive arterial blood pressure monitoring is a common practice in intensive care units (ICUs). The most frequent indication for invasive arterial blood pressure monitoring is for continuous measurement in hemodynamically unstable patients [1]. The radial artery is most commonly used, with the femoral artery being the second choice. One or the other is used in 92% of arterial cannulations [2]. Accuracy of invasive blood pressure monitoring is crucial in evaluating the cardiocirculatory system and adjusting drug therapy for hemodynamic support. However, the best site for catheter insertion is controversial. For some clinicians, the femoral artery is the preferred site because of its lower rate of mechanical (occlusion, accidental loss, thrombosis) and infectious complications [2-4]. The accuracy of peripheral blood pressure compared with central blood pressure measurements has been evaluated by many authors in patients undergoing cardiac surgery [5-12]. Unfortunately, in this setting the population is homogeneous and very different from critically ill patients found in a medical and surgical ICU.

In critically ill patients treated with vasoactive drugs, Dorman and colleagues [13] reported that radial arterial pressure monitoring significantly underestimates central arterial pressure. Insertion of a femoral line allowed a substantial reduction of the infusion rate of vasoactive drugs in these patients [13]. These findings might imply that femoral placement of arterial lines is the gold standard for invasive arterial blood pressure monitoring in shock patients. Nevertheless, that study involved a selected group of patients with postoperative septic shock and only norepinephrine was used as a vasoactive drug. In addition, interchangeability between measurements was not adequately evaluated.

Lack of definitive information in critically ill patients makes it difficult to establish guidelines for daily practice in intensive care. We hypothesize that peripheral and central mean arterial blood pressures are interchangeable in critically ill patients. To test our hypothesis we compare simultaneous measurements of arterial blood pressure in peripheral and central arteries in a heterogeneous population of critically ill patients using formal Bland-Altman analysis [14].

## Materials and methods

### Study population

The study was approved by the Hospital Ethics Committee and the need for informed consent was waived because no additional procedures apart from usual intensive care practice were involved.

Fifty-five critically ill patients admitted to our mixed (medical-surgery) ICU from 16 December 1999 to 22 December 2000 were studied. Inclusion criteria were: clinical indication of invasive arterial pressure monitoring, such as cardiovascular instability, use of intravenous vasoactive agents, and need for frequent sampling of arterial blood [1]; and the need to change the insertion site of the arterial line. Fever and suspicion of catheter-related infection were the main reasons to change the arterial insertion site. The indication was determined following internationally accepted guidelines [15]. Exclusion criteria were: post-cardiac surgery patients; patients with catheter malfunctioning detected by the 'fast flush test' (the pressure in the line was rapidly increased to 300 mmHg by flushing the system with the continuous flow mechanism and the resulting waveform was analyzed to determine the response of the system; ideally, one large and one small oscillation should occur, after which the waveform should be returned to the baseline [16]); patients who needed to be in positions other than the semirecumbent supine; patients with clinical history of peripheral arterial occlusive disease. These two last criteria were based on the possibility of registering artificially or pathologically modified data. Post-cardiac surgery patients were excluded because they are a homogeneous population in which the issue of radial-to-femoral arterial pressure gradient has been well investigated [5-12].

The clinical features of each patient's disease guided indications of invasive arterial blood pressure monitoring and all patients received standard treatments following the guidelines for the pathologies diagnosed or suspected.

Patients were separated into two groups: those receiving high doses of vasoactive drugs (dopamine ≥10 μg/kg/minute or epinephrine or norepinephrine ≥0.10 μg/kg/minute); and those receiving low doses of vasoactive drugs (dopamine <10 μg/kg/minute or epinephrine or norepinephrine <0.10 μg/kg/minute) or no vasoactive. Demographic data (sex and age), APACHE II score [17] at enrollment, number of organ failures (SOFA score) [18], and type (dopamine, epinephrine or norepinephrine) and dose of vasoactive drugs used were recorded.

### Study design

For femoral arteries, 14- or 16-gauge catheters were used (Secalon T 16 G/2.0 × 160 mm Ohmeda, Swindon, Great Britain) and for radial arteries, a 20-gauge catheter was used (Vasculon 2 20 G/32 mm BOC Ohmeda, AB SE-2506, Helsingborg, Sweden). The catheters were inserted with their tips pointing towards the blood flow. Indwelling devices were connected to a continuous-flush transducer system through a rigid plastic tube measuring 120 cm in length in all cases, regardless of whether central or peripheral arteries were used (Becton Dickinson DTX PLUS DT 4812, BD Infusion Therapy Systems, Inc., Sandy, Utah, USA). Both transducers were placed at the same level (right atrium) on a plastic support and zeroed to atmosphere. The arterial blood pressure signals were recorded and displayed on a bedside monitor (Viridia M1205A 24CT, Hewlett Packard, Andover, MA, USA) and the waveforms were simultaneously and permanently registered online. The whole tubing system was flushed with sterile normal saline to eliminate air bubbles and tested for system loss (for instance any kind of fluid leak from the circuit). The monitoring device was connected to a permanent pressurized washing system. Curve characteristics were constantly evaluated using a rapid flush test to rule out occlusion or catheter malposition [16].

The readings over the first five minutes after the insertion of the second catheter were simultaneously recorded, and the mean values for systolic, diastolic, and mean arterial pressures were calculated for both catheters. The data from the entire population were analyzed to determine the global accuracy of the peripheral measurement of mean blood pressure. Next, the two groups were analyzed separately and differences between groups were evaluated to determine the interchangeability of peripheral and central mean arterial blood measurements. We focused the analysis of interchangeability on mean arterial pressure because the tissue perfusion pressure is mainly given by mean arterial pressure rather than by systolic or diastolic pressure.

### Statistical analysis

Data were analyzed using the Bland and Altman method [14]. Bias, precision, and 95% limits of agreement of the simultaneous measurements were calculated [14]. Bias and precision between groups were compared using unpaired *t* tests. Descriptive data are expressed as mean ± standard deviation (SD). Statistical significance was defined as *p *< 0.05.

## Results

The characteristics of the 55 patients are shown in Table 1. The most common reasons for admission were respiratory insufficiency, shock, and postoperative monitoring. Forty patients were classified as receiving high doses of vasoactive drugs and 15 were considered to be receiving low doses. Patients in the high-dose group were receiving dopamine (*n *= 12, doses ranging from 11 to 46 μg/kg/minute), norepinephrine (*n *= 16, doses ranging from 0.11 to 13.5 μg/kg/minute), or epinephrine (*n *= 12, doses ranging from 0.33 to 7.4 μg/kg/minute); 5 patients in this group were also receiving dobutamine simultaneously. Five patients in the low-dose group were receiving dopamine in doses ranging from 3 to 7 μg/kg/minute, one patient was on norepinephrine 0.063 μg/kg/minute, and nine were not receiving any vasoactive drug.

No differences were found in systolic, diastolic, or mean arterial blood pressure measured in the femoral artery versus the radial artery in the entire population or in either of the two groups (Table 2).

**Figure 1:**
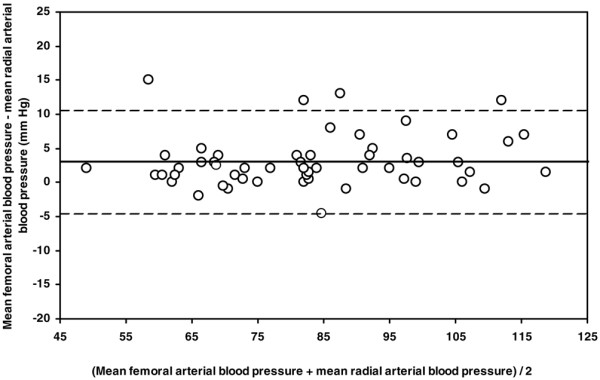
Plot of differences against averages of simultaneous measurements of femoral and radial mean arterial blood pressures. The solid line represents bias (the mean difference between simultaneous measurements). Dotted lines show 95% limits of agreement (bias ± 1.96 standard deviation). The small bias (mean difference between simultaneous measurements) and the narrow 95% limits of agreement suggest the interchangeability of both measurements.

For the whole population, bias (mean difference between simultaneous measurements) ± precision (SD of the difference between those values) of simultaneous femoral and peripheral mean arterial blood pressure measurements was 3 ± 4 mmHg. With these values, the 95% limits of agreement (mean ± 2 SD of the difference between simultaneous measurements) are 16 mmHg (Figure 1). No differences in bias ± precision were found between the high (3 ± 4 mmHg) and low-dose (3 ± 4 mmHg) groups.

## Discussion

The main finding of this study is that central and peripheral mean arterial blood pressures appear to be interchangeable. The 95% limits of agreement of 16 mmHg is not a clinically relevant difference in mean arterial pressure and the two measurements agree regardless of whether patients were receiving vasoactive drugs.

O'Rouke and colleagues [19] have shown that there are no differences in mean arterial blood pressure simultaneously measured in the aorta and radial arteries in healthy volunteers. However, systolic and diastolic arterial blood pressures are higher and lower, respectively, in radial arteries than in the aorta. This phenomenon is known as distal pulse amplification and is due to the characteristics of the vascular tree. Briefly, a pulse waveform entering the aorta is exposed to a sudden impedance change at the capillary level, resulting in a large increment in resistance and producing reflected pulse waveforms. Those waves are added to the following ones, producing higher peaks than the original aortic systolic peak at different distances from the aortic origin. This distal pulse amplification is always present when peripheral vascular resistance is high [19]. In our study we found no evidence of this phenomenon. In fact, systolic, mean, and diastolic pressures were higher in the femoral artery than in the radial artery. Lack of physiological distal pulse wave formation could be due to a vasoactive effect in shock patients. Thus, although vasoactive drugs act mainly on resistance vessels, they also affect conductance vessels, which could alter peripheral arterial blood pressure measurements.

Yazigi and colleagues [20] studied normal volunteers to determine whether radial arterial pressure accurately reflects changes in blood pressure induced by nicardipine. They concluded that peripheral arterial pressure is an accurate measure of central arterial pressure in this setting, and they found no distal pulse amplification.

Invasive arterial blood pressure measurement is a common practice during shock management in the ICU, and the radial artery is the most common site of insertion, followed by the femoral artery. Given the large number of patients requiring high doses of vasoactive drugs during relatively prolonged periods of time and the need to change arterial lines to avoid infectious complications [21-23], it is important to determine whether the measurements are equivalent in alternative cannulation sites.

To our knowledge, this issue has been systematically approached only by Dorman and colleagues [13] in 14 postoperative patients with septic shock receiving high doses of norepinephrine (86 ± 25 μg/minute). A systematic underestimation of mean and systolic arterial blood pressure was found for measurements in the radial artery with respect to the femoral artery. Consequently, this finding allowed the doses of norepinephrine to be decreased and even withdrawn in two patients. After changing the dosage of norepinephrine, differences between mean radial and femoral arterial blood pressures disappeared [13].

Discrepancies between our results and those reported by Dorman and colleagues might be related to different issues. First, there are probably intrinsic differences in the populations studied. The diagnoses of our patients were more heterogeneous (medical and postoperative patients with and without shock) and a broader range of doses of different vasoactive drugs was used. Only 17 (16 in the high dose group and one in the low dose group) patients included in our study were receiving norepinephrine; however, bias and precision between peripheral and central arterial blood pressure was the same in the different groups.

Another source of discrepancy might be the measurement technique used. We tried to minimize variability in the measurement system. For this reason, simultaneous recordings of both pressures were registered on the same monitor using transducers, plastic lines, and washing systems sharing similar features. Nevertheless, the size of the catheters inserted at different sites was different in our study. Although the intravascular portion of the catheter has minimal effect on the accuracy of measurement [16], we cannot rule out the possibility that the pulse wave might be modified by different cannula sizes. Our results might be biased by measurements through smaller catheters in peripheral arteries. However, the small bias found in this study suggests that our results were not influenced by this issue.

Finally, Dorman and colleagues used *t* tests to compare radial and femoral arterial blood pressure measurements; however, when the main issue to be addressed is agreement between different measurements of a variable, the best statistical approach is the Bland and Altman method [14]. There is no definition of the extent to which differences between both measurements might be relevant. Bland and Altman suggested that if the value of the 95% limits of agreement of two methods is not clinically important, they might be interchangeable [14]. The small bias and its narrow standard deviation between peripheral and central arterial blood pressure measurements suggest their interchangeability.

## Conclusion

In this study, peripheral and central measurements of arterial blood pressure showed good agreement regardless of vasoactive drug use. Our results suggest that these two measurements are interchangeable and it is, therefore, not mandatory to cannulate the femoral artery to measure arterial blood pressure, even in critically ill patients receiving high doses of vasoactive drugs.

## Key messages

• 	Femoral and radial mean arterial blood pressures showed good agreement regardless of the use of vasoactive drugs.

• 	Our results suggest that these two measurements are interchangeable.

## Abbreviations

ICU = intensive care unit; SD = standard deviation.

## Competing interests

The authors declare that they have no competing interests.

## Authors' contributions

MAM and EAP participated in the conception and design of the study, in the acquisition analysis and interpretation of the data and drafted the manuscript. AD participated in the conception and design of the study, in the analysis and interpretation of the data, revised the manuscript critically for important intellectual content and gave final approval of the version to be published.

## Figures and Tables

**Table 1:** Characteristics of the study population (*n *= 55)

Characteristic	Vasoactive dose	
	High (*n *= 40)	Low (*n *= 15)

Gender (men:women)	19:21	7:8
Age (years)	68 ± 16	66 ± 15
APACHE II score	24 ± 7	16 ± 7
Number of organ failures	4 ± 2	2 ± 1
Patients on mechanical ventilation (%)	40 (100)	15 (100)
Patients with pulmonary artery catheter (%)	32 (80)	3 (20)
Patients with vasoactive drugs (%)	40 (100)	7 (47)
Patients with dopamine (%)	12 (30)	6 (40)
Patients with epinephrine (%)	12 (30)	0 (0)
Patients with norepinephrine (%)	16 (40)	1 (7)

**Table 2:** Mean, systolic and diastolic arterial pressures in both groups

	MAP (central)	MAP (peripheral)	SAP (central)	SAP (peripheral)	DAP (central)	DAP (peripheral)
Overall (*n* = 55)	85 ± 17	82 ± 17	135 ± 31	126 ± 30	63 ± 14	62 ± 13
High dose (*n* = 40)	85 ± 16	82 ± 15	137 ± 31	124 ± 28	63 ± 13	62 ± 12
Low dose (*n* = 15)	84 ± 20	81 ± 20	130 ± 31	130 ± 33	62 ± 19	60 ± 16

All values are mean ± standard deviation. Overall, entire study population; High dose, high dose vasoactive drug group; Low dose, low dose vasoactive drug group. DAP, diastolic arterial pressure MAP, mean arterial pressure; SAP, systolic arterial pressure.
